# Amino Acids in Health and Disease: The Good, the Bad, and the Ugly

**DOI:** 10.3390/ijms24054931

**Published:** 2023-03-03

**Authors:** Isao Ishii, Madhav Bhatia

**Affiliations:** 1 Department of Health Chemistry, Showa Pharmaceutical University, Tokyo 194-8543, Japan; 2 Department of Pathology and Biomedical Science, University of Otago, Christchurch 8140, New Zealand

The Special Issue “Amino Acid Metabolism and Regulation in Health and Disease 2.0”, edited by us, is the successor of the previous “Amino Acid Metabolism and Regulation in Health and Disease”, edited by Dr. Hieronim Jakubowski (New Jersey Medical School) and us. This Second Issue was opened for submission from 5 May 2021 to 23 October 2022 (the deadline extended by requests), and collected 10 original articles (6 rejected) and 4 comprehensive reviews. The aim of this issue is to highlight novel findings or hypotheses in amino acid metabolism and regulation, especially in terms of health and disease, beyond its fundamental roles as protein constituents and nutrition (e.g., the major nitrogen sources).

## 1. Opposing Roles of Dietary Methionine

The first post came from an unexpected field: methionine (Met) nutrition in “eels”. Recent studies have reported that soybean protein can be used to replace fish meal in aquatic feed; however, Met is the first limiting amino acid in soybean protein. Therefore, Met essential for fish must be obtained from feed. Hu et al. 2021 investigated the impacts of six isonitrogenous and isoenergetic feeds (with 0, 2, 4, 6, 8, 10 g/kg Met) on rice field eel (Asian swamp eel) *Monopterus albus* [1]. They found that Met supplementation increases lipid deposition of the eel in dose-dependent manners, especially in the liver, by regulating hepatic fatty acid metabolism rather than hepatic amino acid contents [1]. Thus, Met supplementation to soybean protein may be also key for the farming of edible eels, including Unagi (*Anguilla japonica*) and Anago (*Conger conger*) in the traditional cuisine of Japan.

Conversely, it is well recognized that Met is also the most toxic amino acid in mammals [2]. Ishii et al. 2022 investigated the impact of Met excess in diets (×1 [0.44%]–×13 [5.72%] Met of the standard rodent diet) on C57BL/6J mice [3]. A high-Met diet for one-week induced a dose-dependent decrease in body weight and an increase in serum Met/homocysteine (Hcy) levels. Similarly, levels of Met and Hcy (but not the other amino acids) were highly elevated in the cerebrospinal fluids of mice. As results, the mice on the 10×Met diet for a week displayed increased anxiety and decreased traveled distances in a conventional open-field test, increased activity to escape from water soaking and tail hanging, while maintaining normal learning/memory activity in a Y-maze test, which are general reflections of negative/positive symptoms and normal cognitive function, similar to bipolar disorder [3]. Therefore, high Met feed might be an easy, quick, and less expensive tool to produce a higher brain dysfunction model in mice, which could be relevant to patients with hypermethioninemia (caused by variants in the *MAT1A*, *GNMT*, or *AHCY* gene) or homocysteinemia (caused by mutations in the *CBS* or *MTHFR* gene).

## 2. Amino Acid-Derived Biomarkers

Two reports arose from the clinical/analytical chemistry field. It is well established that the elevated blood levels of Hcy are an independent risk factor of the cardiovascular disease (CVD). Hcy is known to be metabolized to the thioester Hcy-thiolactone (HTL) in an error-editing reaction during protein biosynthesis when Hcy is erroneously recognized in place of Met by methionyl-tRNA synthetase [4]. Previous studies have demonstrated that Hcy/HTL and formaldehyde (FA), both of which are elevated in blood of aging populations, are implicated in a number of diseases including CVD, Alzheimer’s disease, and diabetes mellitus, and naturally occurring FA undergoes simple, non-enzymatic condensation with Hcy/HTL, producing 1,3-thiasinane-4-carboxylic acid (TCA). Based on these observations, Piechocka et al. 2022 developed an effective analytical tool using gas chromatography coupled with mass spectrometry (GC-MS) for the determination of TCA and detected TCA for the first time in human urine samples [5], although the physiological roles of TCA remain to be elucidated.

Baskal et al. 2022 analyzed biochemical changes using anti-TCR/anti-TNF-α antibody therapy in rat models of human type 1 diabetes [6]. They applied a GC-MS method to measure free and proteinic *N*^ε^-glycated and *N*^ε^-methylated of Lys and all free and proteinic amino acids. Proteins of pancreas, kidney, liver, spleen, and lymph nodes of normoglycemic control, acute diabetic, chronic diabetic and cured rats after the therapy were comparatively analyzed, and most statistical differences between the study groups were observed for spleen, pancreas and kidney, with liver and lymph nodes showing no such differences. In both the pancreas and kidney, the groups differed with respect to proteinic furosine and free *N*^ε^-carboxymethyl lysine. They concluded that those findings underscore the physiological importance of the spleen in this animal model of human type 1 diabetes [6].

## 3. Amino-Acid-Derived Nitric Oxide, Nitrate, and Nitrite

Nitric oxide (NO) is an endogenous potent vasodilator/bronchodilator enzymatically produced from L-arginine (Arg) by constitutive and inducible nitric oxide synthases (NOS; namely, endothelial NOS [eNOS], neuronal NOS [nNOS], and inducible NOS [iNOS]). Nitrate (NO_3_^−^) and nitrite (NO_2_^−^) have been considered as inert end products of endogenous NO metabolism; however, some studies show that these anions can be recycled in vivo to from NO, representing an important alternative source of NO to the classical Arg/NOS pathway, in particular in hypoxic states [7]. Hanusch et al. 2022 measured the blood and urinary levels of the Arg/NOS pathway metabolites in 130 of 302 participating children with atopic dermatitis and/or bronchial asthma in several German hospitals [8]. They found that the Arg/NOS pathway was activated in one or two atopic diseases (e.g., higher plasma NO_3_^−^ and NO_2_^−^ independent of their severity) and the systemic NO synthesis was upregulated in children with atopic diseases perhaps due to their higher systemic inflammatory activity [8].

A single report was from sport medicine. Supplementation of inorganic NO_3_^−^ has been known to increase endogenous NO levels, independent of NOS and presumably via its reduction to NO_2_^−^, which is assumed to increase the physical power during high-intensity exercise. NO_3_^−^ is also known to enhance the enzyme activity to hydrolyze asymmetric dimethylarginine (ADMA), an endogenous NOS inhibitor. Based on this background, Tsikas et al. 2022 investigated the effects of 0.14 mmol/kg/day NaNO_3_ ingestion (in comparison to the same dose of NaCl) on the amino acid homeostasis and oxidative stress in 17 healthy volunteers (9 for NaNO_3_ and 8 for NaCl) [9]. The plasma concentrations of NO_3_^−^ (×7.4), NO_2_^−^ (×1.67), homoarginine (×1.32), ornithine/citrulline (×1.06), and glutamate/glutamine (×1.20) were higher whereas those of sarcosine, tyrosine (×0.864), phenylalanine (×0.923), tryptophan (Trp; ×0.921), and malondialdehyde (MDA; ×0.890) were lower in the NaNO3 group compared to the NaCl group, suggesting that the NaNO_3_ supplementation increased the Arg/glycine (Gly) amidinotransferase (AGAT)-catalyzed homoarginine synthesis and decreased the *N*-methyltransferase-catalyzed synthesis of guanidinoacetate, the precursor of creatine. These results illustrate the possible systemic positive and negative impacts of NaNO_3_ supplementation for the purpose of muscle strengthening [9].

## 4. Val/Ile Supplementation to Protein-Restricted Pigs

Two reports in the field of animal husbandry were posted from the Pezeshki’s group [10,11]. High protein diets have been criticized to be used for pigs due to their negative impact on the environment, diet cost, post-weaning diarrhea, and human/animal health. Therefore, slightly low protein diets with less than 4% reduced crude protein plus first four limiting amino acids (lysine (Lys), Met, threonine, and Trp) are used to improve nitrogen secretion, diarrhea, and gut health with no negative impacts of their growth performance. However, very low protein (VLP) diets with more than 4% reduced crude protein are known to reduce their growth performance. The first study examined the effects of valine (Val), isoleucine (Ile) or both supplementation on the growth performance of VLP-fed pigs containing the first four limiting amino acids and assessed whether that is accompanied by alterations in blood metabolomics, gut development, and hepatic IGF-1 expression [11]. Habibi et al. 2022 demonstrated that the combination of Val and Ile improved food intake, gut development, and hepatic IGF-1 expression, and plasma metabolomics profiles. The following study examined the impacts of the supplementation of Val above the National Research Council (NRC) level and/or Ile at the NRC levels to VLP-fed pigs [10]. Goodarzi et al. 2022 found that, while both VAIL (supplemented with Val above and Ile at NRC levels) and VA (supplemented with Val above NRC levels) groups completely recovered the inhibitory effects of VLP diets on food intake, only VAIL partially recovered the negative effects of VLP diets on growth performance, possibly by altering their gut microbiota (e.g., higher abundance of colonic *Actinobacteria*, *Enterococcus*, and *Enterobacteriaceae*) [10].

## 5. Homoarginine Application in Rat Models of Cardiomyopathy

Low circulating (and low excretory) concentrations of homoarginine (hArg) have been shown to be associated with worse cardiovascular outcomes (e.g., acute ischemic stroke) and mortality [12]. Plasma hArg concentration is regulated by AGAT that catalyzes (1) the formation of guanidinoacetate (and ornithine) from Arg and Gly and (2) the formation of hArg (and ornithine) from Arg and Lys. Guanidinoacetate is further metabolized to creatine by guanidinoacetate *N*-methyltransferase (GAMT). A genome-wide association study revealed that three SNPs in the AGAT gene are significantly associated with low plasma hArg concentration. hArg was barely detectable in AGAT-knockout mice that showed reductions in body weight and adiposity, and improved glucose tolerance and creatine levels, whereas GAMT-knockout mice in which AGAT is upregulated displayed creatine deficiencies and increased levels of hArg. Based on such background, Tsikas et al. 2022 investigated free and proteinic amino acid profiles in the liver, kidney, heart, and lung of hArg-administered rat models of isoprenaline-induced takotsubo cardiomyopathy [13]. From their results, the authors conclude that Lys and Arg are major metabolites of exogenously administered hArg and kidneys and heart seem to play a major metabolic role for hArg. This study also showed that free hArg and N-methylglycine are positively associated with each other. Therefore, this study showed remarkable changes in free and proteinic amino acids in different organs in the rat models of cardiomyopathy [13].

## 6. Essential Amino Acids and Resistance Exercise Training

Jang et al. 2022 investigated the impact of the combination of an essential amino acid diet and resistance exercise training (RET) against side effects by the use of dexamethasone in mice [14]. Dexamethasone is extensively used in the medial field to treat a variety of inflammatory diseases, but its chronic use is known to cause severe side effects such as impaired proteostasis and glucose metabolism, leading to muscle mass loss and a decrease in muscle strength and endurance. They demonstrated that essential amino acids supplementation and RET synergistically protects muscle from the dexamethasone-induced side effects, at least in part through positively affecting the rate of myofibrillar protein synthesis, neuromuscular junction stability, preservation of muscle fiber type shifting to fast glycolytic fiber, and mitochondrial biogenesis. Notably, the combination therapy also completely restored impaired insulin sensitivity through the use of dexamethasone treatment.

## 7. Four Seminal Reviews

Kumar et al. 2021 overviewed the roles of hydrogen sulfide (H_2_S, a gasotransmitter with potent/diverse physiological functions that enzymatically derives from sulfur-containing amino acids), substance P (an eleven-amino-acid neuropeptide involved in vasodilation, inflammation, and pain), and adhesion molecules, including ICAM-1, VCAM-1, and selectins, in acute pancreatitis, one of the main reasons for hospitalization amongst gastrointestinal disorders [15]. They concluded that hydrogen sulfide and substance P interact with each other and contribute to the development and progress of acute pancreatitis, at least in parts by modulating the expression of adhesion molecules.

Belostotsky et al. 2022 provided a review on the catabolism of hydroxyproline (Hyp), one of the most prevalent amino acids (roughly 4%) in animal proteins that is produced by hydroxylation of proline by prolyl hydroxylase [16]. Hyp is a major component of collagen, comprising ~13.5% of mammalian collagen. Because Hyp cannot be re-incorporated to proteins, it is catabolized following protein degradation into two deleterious intermediates: glyoxylate and hydrogen peroxide, which need to be immediately converted. They discussed about (1) different catabolism between herbivores and carnivores, (2) primary hyperoxaluria (PH), a genetic disorder associated with renal dysfunction resulting from excessive glyoxylate conversion to oxylate by lactate dehydrogenase, and (3) new PH-targeted siRNA therapy [16].

Morland et al. 2022 summarized the roles of *N*-acetyl-aspartyl-glutamate (NAAG) in brain health and disease [17]. NAAG is the most abundant dipeptide selectively localized to the brain that plays a neuromodulatory roles in glutamatergic synapses. They first summarized its target receptors (e.g., mGluR3), its role as an anterograde or retrograde neurotransmitter, its synthesis and degradation, and its roles in cognition. Thereafter, they explained its pathophysiological roles in disease conditions such as neurodegenerative disorders (such as Alzheimer’s disease and Parkinson’s disease), epilepsy, stroke, traumatic brain injury, pain, and schizophrenia. Because neuroprotective NAAG is rapidly degraded by glutamate carboxypeptidase II (GCPII) and III (GCPIII), they finally commented on the future aspects of clinical application of GCPII inhibitors [17].

Cuomo et al. 2022 described altered branched-chain amino acid (BCAA; namely, Val, Ile, and leucine (Leu)) metabolism in disease conditions, including type 2 diabetes, obesity, CVD, and nonalcoholic fatty liver disease (NAFLD) [18]. Several lines of evidence suggest that diets rich in BCAAs are associated with those metabolic diseases, whereas diets low in BCAAs are generally promote metabolic health. They argued about the involvement of circulatory Gly and adiponectin levels, gut microbiota, and altered gene expression as the rheostat to balance metabolic health and disease.

**Closing Remarks**—This Special Issue represents a collection of original articles and review papers from a wide range of scientific fields and demonstrates the importance of amino acids as the most fundamental signaling molecules. In addition to playing a key role on health and disease, amino acids, in biology, are also precursors for gaseous mediators–NO and H_2_S, which are produced as a result of metabolism of Arg and cysteine, respectively. These two gases also make a vital contribution to the biological process. Recent research has unraveled many mysteries on the role of amino acids in health and disease. We hope that the articles published in this Special Issue will form the basis for more groundbreaking research in times to come.
